# Placental abruption and perinatal mortality in twins: novel insight into management at preterm versus term gestations

**DOI:** 10.1007/s10654-024-01171-z

**Published:** 2024-11-22

**Authors:** Rachel Lee, Justin S. Brandt, Cande V. Ananth

**Affiliations:** 1 Division of Epidemiology and Biostatistics, Department of Obstetrics, Gynecology, and Reproductive Sciences, Rutgers Robert Wood Johnson Medical School, New Brunswick, NJ USA; 2 Division of Maternal-Fetal Medicine, Department of Obstetrics and Gynecology, NYU Grossman School of Medicine, New York, NY USA; 3 Cardiovascular Institute of New Jersey, Rutgers Robert Wood Johnson Medical School, New Brunswick, NJ USA; 4 Department of Biostatistics and Epidemiology, Rutgers School of Public Health, Piscataway, NJ USA; 5 Department of Medicine, Rutgers Robert Wood Johnson Medical School, New Brunswick, NJ USA

**Keywords:** Placental abruption, Perinatal mortality, Preterm delivery, Causal mediation analysis

## Abstract

Twins suffer a disproportionately higher burden of adverse perinatal outcomes than singletons. However, the degree to which preterm delivery shapes the relationship between abruption and perinatal mortality in twins is unknown. Through causal mediation decomposition, we examine how preterm delivery mediates the effect of abruption on perinatal mortality among twins using the US-matched multiple birth data (1995–2000). We estimated the hazard ratio (HR) from Cox models with gestational age as the timescale. We decomposed the total effect (TE) into counterfactual natural direct (NDE) and natural indirect (NIE) effects. 557,220 matched twin births, 1.3% (*n* = 7032) resulted in abruption with higher perinatal mortality rates than non-abruption births (143 versus 36 per 1000 births, respectively) and a 4.53-fold (95% confidence interval [CI]: 4.23, 4.82) increased hazard of perinatal mortality. HRs for NDE and NIE were 3.05 (95% CI: 2.84, 3.24) and 1.49 (95% CI: 1.49, 1.47, 1.50), respectively, and the proportion mediated (PM) was 41%. PM increased as the gestational age at delivery decreased. Associations persisted after correction for unmeasured confounders. The best strategies to improve perinatal delivery are delivery when abruption complicates twin pregnancies at term gestations and expectant management (avoiding early preterm delivery), if feasible, when abruption complicates twin pregnancies at preterm gestations.

**Supplementary Information:**

The online version contains supplementary material available at 10.1007/s10654-024-01171-z.

## Introduction

Placental abruption is the premature detachment of the placenta from the uterine wall before the delivery of a fetus. This condition complicates 0.5–1.2% of all pregnancies and is associated with painful vaginal bleeding that often necessitates urgent delivery [1]. Pregnancies complicated by abruption are at increased risk of preterm delivery, stillbirth, and perinatal mortality [2]. Perinatal mortality is almost 15 times higher in abruption (119.2 per 1000) compared to non-abruption (8.2 per 1000) births in singletons [3]. Multiple gestations may further compound abruption risk as twin pregnancies carry a 1.5 to 2-fold higher risk of abruption than singletons [4].

Twin births in the United States (US) have increased substantially over the last decades from 19.2 in 1980 [5] to 31.2 per 1000 total births in 2021 [6]. This upward trend has been attributed to increased use of assisted reproduction technology (ART) [7] and a shift towards an older age at conception when multifetal gestations are more likely to occur naturally [8]. Risks of pre-labor rupture of membranes, spontaneous preterm delivery, neonatal morbidity, and perinatal mortality are substantially higher in twin versus singleton births [9]. Perinatal mortality is 3–4 fold higher in twins compared to singletons [9, 10], with 62% of US twin births in 2021 delivered preterm [6] and 25–47% of twin births complicated by fetal growth restriction [11]. Preterm delivery risks are 1.5 times higher among twin pregnancies with versus without abruption [12].

Gestational age is a powerful predictor of perinatal survival [13, 14], and abruption rates decline with advancing gestation [15]. The management of pregnancies complicated by abruption often involves obstetrical interventions (either labor induction or pre-labor cesarean). This, in turn, results in increased risks of clinician-initiated deliveries at preterm gestations. Investigators have synthesized these observations by evaluating how preterm delivery mediates the association between abruption and perinatal mortality in singleton births [16]. In an analysis of 26 million US singleton births (1995–2002) that compared abruption versus non-abruption singleton pregnancies, 28% of the increased perinatal mortality associated with abruption was mediated through preterm delivery [15]. Similar data among twins is lacking. Through a causal mediation approach, we evaluate the role of preterm delivery (< 37, < 34, and < 32 weeks) as a mediator on abruption and stillbirth, neonatal, and perinatal mortality associations in twin gestations.

## Methods

We used the National Center for Health Statistics (NCHS) Matched Multiple Birth Data Set (1995–2000), which allows for analysis of characteristics specific to the multiple sets in live births and fetal deaths in the US [17]. Abruption status was determined on the birth certificate form. Preterm delivery was examined at clinical gestational ages < 37, <34, and < 32 weeks. We assessed the risks of abruption and preterm delivery on stillbirth (fetal death after 20 weeks gestation), neonatal mortality (0–27 days after delivery), and perinatal mortality defined as stillbirth and neonatal deaths. The cohort consisted of matched twins ≥20 weeks in gestational age. We excluded triplets or higher order multiple gestation pregnancies, missing gestational age, gestational age less than 20 weeks, missing abruption status, and unmatched twin births (Supplemental Fig. 1).

### Statistical analyses

We assessed the rates of perinatal mortality among abruption and non-abruption deliveries. We fit a Cox proportional hazard model to calculate the unadjusted hazard ratio (HR) and 95% confidence interval (CI) of abruption and perinatal mortality association with gestational age as the time scale.

We undertook a causal mediation analysis based on the theory of counterfactuals in the setting of a time-to-event analysis [15]. We estimated the adjusted HR and 95% CI fitted by a Cox proportional hazards regression model with gestational age as the time scale. In this model, we treated abruption as the exposure, mortality as the outcome, and preterm delivery as the mediator. We used gestational age as the timescale to remove heterogeneity in mortality risks across gestational ages and to model the instantaneous hazards and latency between birth and the day of death.

### Causal decomposition

**Fig. 1:**
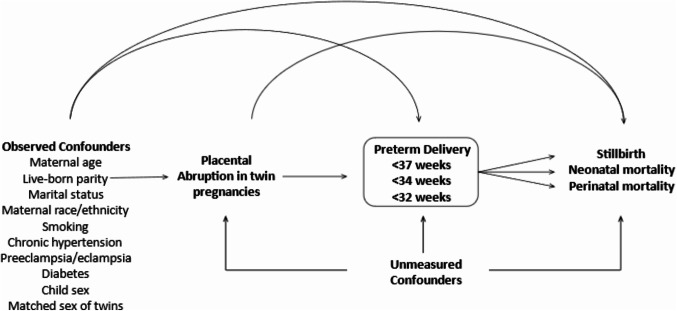
Simplified directed acyclic graph showing the relationship between placental abruption and perinatal mortality with preterm delivery as the mediator: US Matched Multiple Birth File, 1995–2000

Based on a counterfactual framework, we decomposed the total effect (TE) as the overall causal effect of abruption on the perinatal mortality association into two components: (i) a natural direct effect (NDE), defined as the counterfactual estimate of abruption’s direct effect on mortality independent of preterm delivery; and (ii) a natural indirect effect (NIE) defined as the counterfactual estimate of the effect of abruption on mortality that operates through preterm delivery. The proportion mediated (PM) is the proportion of TE of the abruption-perinatal association explained or accounted for by the mediator [18] and derived as PM = NIE/TE [19]. All 95% CI estimates were based on 1000 bias-corrected accelerated bootstrap resampling methods. The causal structure of the associations is shown in the Directed Acyclic Graph (DAG, Fig. 1). All analyses were conducted in R (Version 4.2.2; Boston, MA, USA) using the package *CMAverse* [20].

Several assumptions are involved in causal decomposition, including the no-unmeasured confounding assumptions of the exposure-outcome, mediator-outcome, and exposure-mediator relations and the exposure that confounds the mediator-outcome relation [15]. We implemented a bias analysis without assumptions on unmeasured confounders. We obtained a conservative estimate of the true causal effect by dividing the observed risk and 95% CI by the joint bounding factor [21]. The bounding factor measures the strength of confounding between the exposure and the outcome resulting from the confounder. This implies that the exposure-unmeasured confounder (EU) HR and unmeasured confounder-outcome (UD) HR would have to be as strong as the observed hazard ratio [22]. The joint bounding factor was derived as (HR_EU_ x HR_UD_)/(HR_EU_ + HR_UD_ -1).

### Missing data

To account for missing data in covariates, we imputed the data 10 times, assuming that the missing data were ‘missing at random.’ Based on Rubin’s principle, we pooled the effect estimates across the ten imputed data sets [23].

### Covariates

We adjusted all models for the potential confounding effects of maternal age (categorized in 5-year groups < 20, 20–24, 25–29…≥40), live-born parity (1,2, ≥3), marital status (single, married), race/ethnicity (white, black, other), smoking status (smoker, non-smoker), chronic hypertension, diabetes, preeclampsia/eclampsia, child sex (male, female), and sex of twins (matched females, matched males, mixed sex).

### Sensitivity analysis

We also compared the variance of the causal effects with and without clustering of twin sets in the unadjusted Cox proportional hazards regression models due to limitations in *CMAverse* to account for clustering.

## Results

### Demographic characteristics

Of 557,220 matched twin births, 1.3% (*n* = 7032) births were complicated by abruption (Supplemental Table 1). The risk of abruption increased with maternal age, live-born parity, single marital status, smoking, chronic hypertension, and decreasing gestational age. Over half (56.6%) of all twin births were delivered preterm (< 37 weeks). About half of all twin births complicated by abruption were delivered by 32 weeks; in contrast, about half of non-abruption births were delivered by 36 weeks (Supplemental Fig. 2).

### Abruption and perinatal mortality risk

**Fig. 2:**
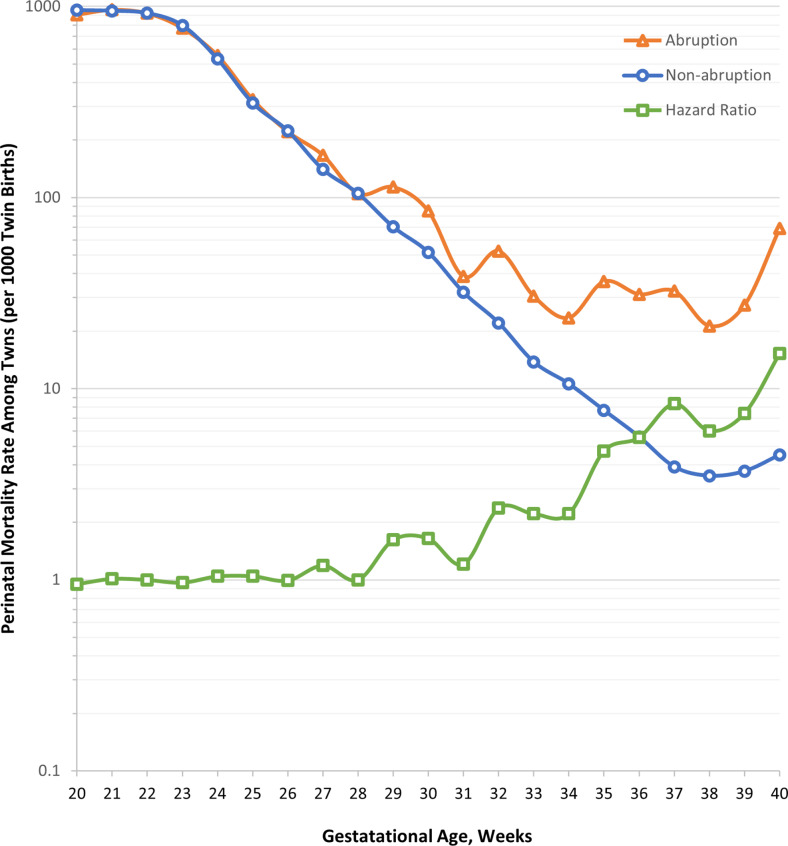
Perinatal mortality rates (per 1000 twin births) among non-abruption and placental abruption births and hazard ratio of mortality by gestational age, US Matched Multiple Birth File, 1995–2000

**Fig. 3:**
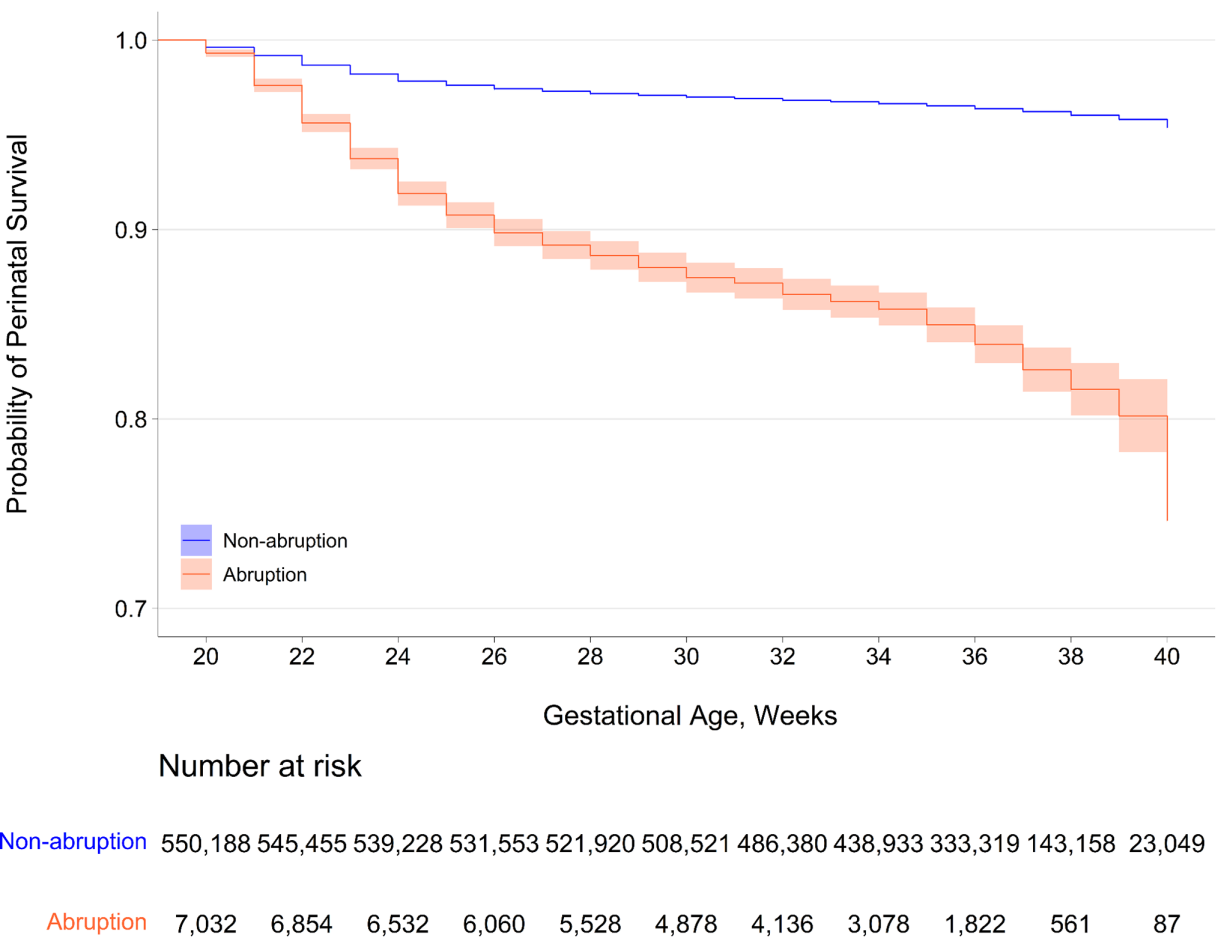
Kaplan-Meier curve showing the probability of perinatal survival among abruption and non-abruption births by gestational age: US Matched Multiple Birth File, 1995–2000

Stillbirth, neonatal mortality, and perinatal mortality rates were higher in abruption than in non-abruption births (Table 1). The risk of perinatal mortality was higher among abruption than non-abruption births (unadjusted HR 4.53, 95% CI 4.26, 4.83). Perinatal mortality risk per 1000 births decreased with gestational age until 38 weeks for both abruption and non-abruption births, but the risk of mortality increased with gestational age for abruption births compared to non-abruption births after 28 weeks (Fig. 2). The Kaplan-Meier curve depicts a worse probability of perinatal survival in abruption births than in non-abruption births (Fig. 3).

**Table 1:** Perinatal mortality rates among twin births in the non-abruption and placental abruption groups: US Matched Multiple Birth File, 1995–2000

Perinatal mortality (per 1000 births)	No abruption		Placental abruption		Excess risk 95% CI	HR (95% CI)
	Total births	Number of deaths (Risk per 1000)	Total births	Number of deaths (Risk per 1000)		
Stillbirth	550 188	8082 (15)	7032	418 (59)	45 (39–50)	4.87 (4.41–5.37)
Neonatal mortality	542 106	11 972 (22)	6614	587 (89)	67 (60–74)	4.48 (4.12–4.87)
Perinatal mortality	550 188	20 054 (36)	7032	1005 (143)	106 (98–115)	4.53 (4.26–4.83)

The stillbirth rate was estimated as the number of twin fetal deaths at ≥20 weeks over the total number of twin births

The neonatal mortality rate was estimated as the number of twin deaths up to 27 days over the number of twin live births

Perinatal mortality included the number of fetal deaths plus neonatal deaths among twins over the number of total twin births

### Mediation through preterm delivery

The total effect HR for abruption and perinatal mortality association was 4.53 (95% CI 4.23, 4.82). NDE HR of 3.05 (95% CI 2.84, 3.24) was higher than NIE HR of 1.49 (95% CI 1.49, 1.47, 1.50) for preterm delivery (< 37 weeks). In contrast, the NDE was lower than the NIE at earlier gestational ages (< 34 and < 32 weeks) (Table 2). Mortality PM through preterm delivery (< 37 weeks) for stillbirth, neonatal mortality, and perinatal mortality was 41%, 43%, and 42%, respectively. In comparison, PM through preterm delivery (< 34 weeks) substantially increased (82%, 84%, and 82%, respectively) and more than doubled at preterm delivery (< 32 weeks) (94%, 95%, 94%, respectively).

**Table 2:** Adjusted hazard ratio (95% confidence interval) of the natural direct and natural indirect effects mediated through preterm delivery of the association between placental abruption and perinatal mortality among twin births: US Matched Multiple Birth File, 1995–2000

	Natural direct effect	Natural indirect effect	Total effect	Mortality proportion mediated through preterm delivery
	HR_NDE_ (95% CI)	HR_NIE_ (95% CI)	HR_TE_ (95% CI)	% (95% CI)
***Preterm delivery < 37 weeks***				
Stillbirth	3.18 (2.87–3.50)	1.48 (1.47–1.50)	4.73 (4.27–5.23)	41 (40, 43)
Neonatal mortality	2.79 (2.59-3.00)	1.49 (1.48–1.50)	4.16 (3.86–4.46)	43 (42–45)
Perinatal mortality	3.05 (2.84–3.24)	1.49 (1.47–1.50)	4.53 (4.23–4.82)	42 (41–43)
***Preterm delivery < 34 weeks***				
Stillbirth	1.63 (1.46–1.80)	2.72 (2.66–2.78)	4.44 (3.96–4.91)	82 (79–85)
Neonatal mortality	1.50 (1.40–1.62)	2.72 (2.66–2.77)	4.07 (3.80–4.40)	84 (82–86)
Perinatal mortality	1.60 (1.51–1.71)	2.72 (2.66–2.77)	4.37 (4.10–4.66)	82 (80–84)
***Preterm delivery < 32 weeks***				
Stillbirth	1.18 (1.05–1.31)	3.48 (3.38–3.58)	4.10 (3.65–4.61)	94 (91–98)
Neonatal mortality	1.15 (1.07–1.23)	3.48 (3.38–3.57)	4.02 (3.69–4.31)	95 (93–97)
Perinatal mortality	1.20 (1.13–1.29)	3.48 (3.38–3.58)	4.18 (3.90–4.51)	94 (92–96)

All models were adjusted for the confounding effects of maternal age, parity, marital status, race/ethnicity, smoking status, chronic hypertension, diabetes, preeclampsia/eclampsia, child sex, and matched sex of twins

All 95% confidence interval estimates were based on bootstrap resampling with 1000 replications

### Sensitivity analysis

**Fig. 4:**
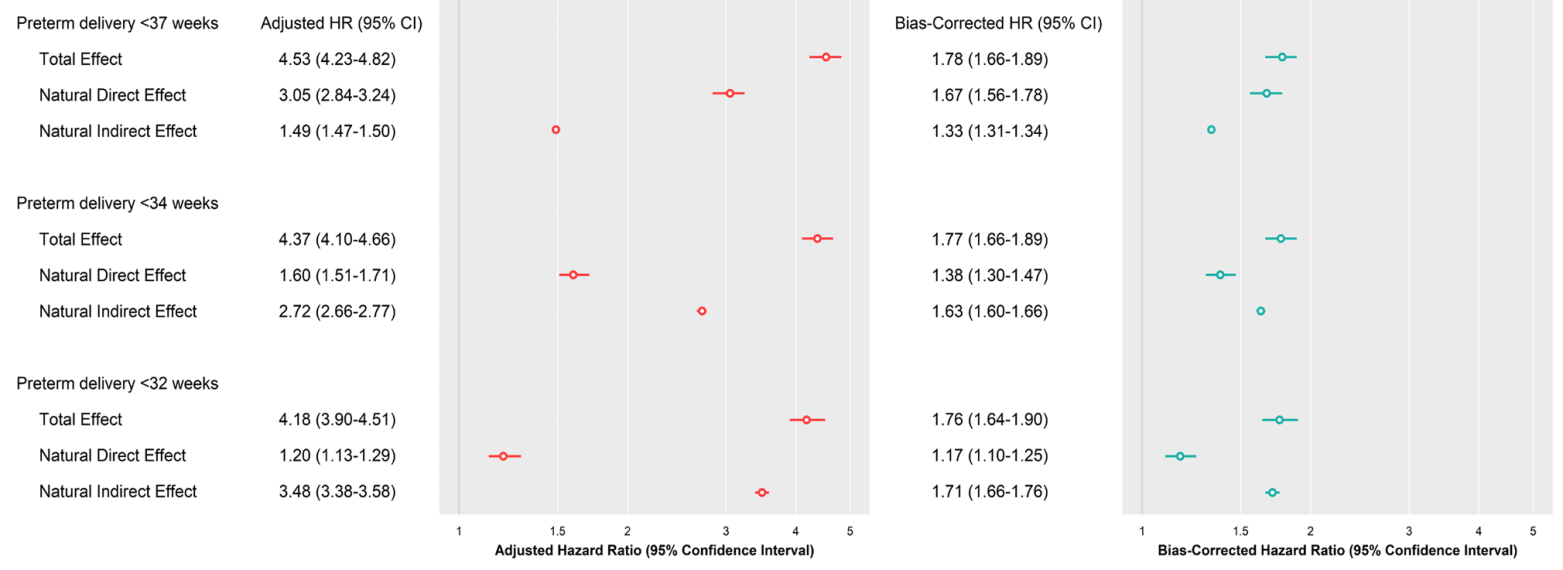
Decomposed effects of preterm delivery on abruption and perinatal mortality association: US Matched Multiple Birth File, 1995–2000

In the sensitivity analysis, the true causal estimates of NDE, NIE, and TE of preterm delivery on abruption and perinatal mortality are attenuated (Fig. 4), but the PM through preterm delivery (< 37, < 34, and < 32 weeks) remained high (Table 3). The confidence interval was wider after accounting for the clustering of twin sets, while the hazard ratios remained unchanged (Supplemental Table 2).

**Table 3:** Bias-corrected hazard ratios for direct and indirect effects (mediated through preterm delivery at < 37, <34, < 32 weeks) of the association between placental abruption and mortality risk, US matched multiple birth file, 1995–2000

	Natural direct effect	Natural indirect effect	Total effect	Mortality proportion mediated through preterm delivery
	HR_NDE_ (95% CI)	HR_NIE_ (95% CI)	HR_TE_ (95% CI)	% (95% CI)
***Preterm delivery < 37 weeks***				
Stillbirth	1.69 (1.52–1.86)	1.32 (1.32–1.34)	1.79 (1.61–1.98)	74 (68–81)
Neonatal mortality	1.64 (1.52–1.77)	1.33 (1.31–1.34)	1.76 (1.63–1.89)	76 (71–80)
Perinatal mortality	1.67 (1.56–1.78)	1.33 (1.31–1.34)	1.78 (1.66–1.89)	75 (71–79)
***Preterm delivery < 34 weeks***				
Stillbirth	1.39 (1.24–1.53)	1.63 (1.60–1.67)	1.77 (1.58–1.96)	92 (85–100)
Neonatal mortality	1.33 (1.24–1.44)	1.63 (1.60–1.66)	1.75 (1.64–1.90)	93 (88–97)
Perinatal mortality	1.38 (1.30–1.47)	1.63 (1.60–1.66)	1.77 (1.66–1.89)	92 (88–96)
***Preterm delivery < 32 weeks***				
Stillbirth	1.15 (1.03–1.28)	1.71 (1.66–1.76)	1.76 (1.56–1.97)	98 (89–100)
Neonatal mortality	1.13 (1.05–1.21)	1.71 (1.66–1.76)	1.75 (1.61–1.88)	98 (94–100)
Perinatal mortality	1.17 (1.10–1.25)	1.71 (1.66–1.76)	1.76 (1.64–1.90)	97 (93–100)

## Discussion

### Principal findings

In this study of US twin births, we found that births complicated by abruption are delivered at earlier gestational ages than non-abruption births. The risk of perinatal mortality decreases with advancing gestational age but is higher in abruption compared to non-abruption births across all gestational ages greater than 27 weeks. Preterm delivery at < 34 and < 32 weeks mediates most of the association between placental abruption and perinatal mortality in twins, while preterm delivery at < 37 weeks mediates this association moderately. The bias-corrected analyses for unmeasured confounding show a high proportion mediated at < 37 weeks. This underscores the consistent observation that the proportion mediated by preterm delivery increases with delivery at earlier gestational ages. These findings have important clinical significance, suggesting that strategies to minimize perinatal mortality for twins complicated by abruption at term is delivery, but expectant management is the preferred approach, when feasible, at preterm gestations.

### Possible pathways

In twin births, about 60% of the increased perinatal mortality risk associated with placental abruption is not explained by preterm delivery (< 37 weeks). This suggests preterm delivery alone at < 37 weeks is not driving the increased perinatal mortality risk. Since over 50% of twin births are delivered preterm, one possible pathway for the direct effect of abruption on perinatal mortality among term deliveries involves fetal hypoxia when oxygen transport is either diminished in cases of partial abruption or completely ceased in the setting of total abruption [15]. Interestingly, 82–94% of the abruption-perinatal mortality association was explained by preterm delivery (< 34 and < 32 weeks), suggesting early delivery (< 34 and < 32 weeks) is more detrimental in twins compared to early delivery (< 28 weeks) in singletons (PM = 46.7%) [15]. Uterine overcrowding [24] and overdistension, unequal distribution of shared placental mass, congenital anomalies, congenital infection, or a combination of multiple factors may contribute to restricted fetal growth among twins. It may contribute to these observed differences [12].

### Clinical implications

With rates of preterm delivery increasing by 4% from 2007 (10.1%) to 2021 (10.5%) in the US, the highest reported since 2007 [25], and contributing to the second leading cause of infant mortality in 2021 [26], clinicians should avoid very early preterm delivery among abruption births, especially among twins. The American College of Obstetricians and Gynecologists (ACOG), in collaboration with the Society for Maternal-Fetal Medicine (SMFM), updated their guidelines in 2021 for medically indicated late-preterm and early-term deliveries for multiple gestation pregnancies with and without complications. In their committee opinion, the authors made no recommendations for pregnancies complicated by abruption due to a lack of data [8, 27]. Our study provides data that should inform clinical management.

The results of our study suggest that strategies to avoid iatrogenic prematurity, such as intensive fetal surveillance, corticosteroids, blood transfusions, and hospitalization, are likely to improve perinatal outcomes among twin gestations complicated by preterm abruption [1, 8, 28]. This strategy of expectant management, when feasible, allows twins to reach later gestational ages with improved neonatal outcomes and reduces the compounding risks of preterm delivery on perinatal mortality. In situations such as partial abruption, where there may be clinical uncertainty about the best approach (preterm delivery or expectant management), the latter approach improves survival in preterm gestations. This management approach is similar to that of pregnancies complicated by fetal growth restriction and cancer. The Growth Restriction Intervention Trial was designed to guide the management of fetal growth restriction. Following the publication of the trial, ACOG/SMFM concluded that uncomplicated fetal growth restriction should not be delivered before 37 weeks [29]. As more people have been treated with chemotherapy in pregnancy, preterm delivery has emerged as a more significant driver of adverse neonatal outcomes than exposure to cancer or chemotherapy [30]. In situations where there is stable maternal status but clinical uncertainty about whether to proceed with delivery, we should add preterm abruption to other clinical entities, including fetal growth restriction and cancer, where expectant management is preferred to clinician-initiated preterm delivery.

For twin births complicated by term abruptions, immediate delivery is recommended since perinatal mortality risks increase with gestational age beyond 38 weeks, and abruption complications could further risk perinatal death [31] as well as maternal morbidity. In these cases, medical optimization followed by induction of labor (or cesarean delivery, if indicated) may reduce the risk, as it appears that exposure to abruption in the term period is the primary driver of risk [1].

### Strengths

One strength of this study was the novelty of using survival analysis with gestational age as the time axis rather than an independent variable due to the temporal nature and lack of exchangeability of gestational age to determine total, direct, and indirect effects in perinatal research [32] and as observed in our study. This population-based study incorporated all twin deliveries in the US, which allowed for the generalizability of the results. With the matched multiple births data, we could account for the sex of twins as a proxy for chorionicity (matched sexes reflecting monochorionic gestations and mixed sexes reflecting dichorionic gestations). Two validation studies of birth certificate data in New York and New Jersey show high specificity (100% and 99.8%, respectively) and moderate abruption sensitivity (67% and 28.5%, respectively) [33, 34]. Although abruption cases may be underreported, there are few false positives. Corrections for unmeasured confounding bias demonstrate that observed associations persist, suggesting preterm delivery is an important mediator in the relationship between abruption and preterm delivery. Our findings are in agreement with many studies that show most perinatal mortality in twins is due to extreme preterm delivery [28, 31, 35–38] and that twin births are predisposed to worse outcomes such as preterm delivery, neurological impairments, a 5-fold risk of stillbirth, and 7-fold neonatal mortality than singletons [39–41]. This could have profound effects later in life because twins have increased risks of long-term cardiac, respiratory, neurologic, infectious, and malignancy complications compared to singletons due to the dangers of being born earlier [42].

### Limitations

Confounding bias is likely due to income, BMI, assisted reproduction technology (ART), and chorionicity (these variables were unavailable in the database). Further studies are needed since ART-conceived twins carry increased risks of preterm delivery compared to spontaneously conceived twins [43], and ART-conceived singleton pregnancies have increased risks of perinatal mortality, preeclampsia, placenta previa, placental abruption, gestational diabetes, and preterm delivery compared to pregnancies conceived spontaneously [44]. More research is needed to assess the mediation effects of clinician-initiated preterm delivery compared to spontaneous preterm delivery on abruption and perinatal mortality [45]. The impact of clinician-initiated preterm delivery compared to spontaneous preterm delivery on neonatal mortality varies among countries. A retrospective population-based study in Slovenia from 2013 to 2018 following 9,200 deliveries (6,252 spontaneous births and 2,948 clinician-indicated) showed an increased risk of neonatal mortality in clinician-induced preterm delivery [46]. while US population-based studies showed the greatest reduction in perinatal mortality occurred among indicated preterm delivery [47–49] and a Swedish retrospective study from 1999 to 2008 showed no impact of preterm delivery type before neonate reaches < 27 weeks of gestation on perinatal mortality [50]. Finally, though it represents one of the largest cohorts of twin births, this data is almost 25 years old. Further investigation is warranted to understand how advances in neonatal care such as periviabilty management [51, 52], neuro-prophylaxis with magnesium sulfate [29], and steroids administration [53] and changes in ART practices [54] could impact the observed mediating effects of preterm delivery on the abruption-perinatal mortality association.

## Conclusions

There are clinical cases in which optimal management is uncertain in the presence of abruption, and clinicians may debate whether to proceed with delivery (with goals of avoiding stillbirth and minimizing morbidly from obstetrical hemorrhage) versus expectant management (sometimes giving multiple blood transfusions and requiring prolonged surveillance in the inpatient settings). Preterm delivery mediates most of the association between placental abruption and perinatal mortality in twins, especially at earlier gestational ages. Therefore, our data suggests that preterm delivery of twins due to abruption complications should be avoided, especially after very preterm gestations, when feasible. Among twin pregnancies complicated by abruption at term, however, delivery appears to be the best approach. While our data utilized a historical cohort that is among the largest of matched twin deliveries with abruption, the results of our study have important clinical implications and underscore the need for additional research in a contemporary cohort that may further refine delivery guidance in modern settings.

## Electronic Supplementary Material

Below is the link to the electronic supplementary material.

Supplementary Material 1
