# Supplementary material for: Placental abruption and perinatal mortality in twins: novel insight into management at preterm versus term gestations
Source: Eur J Epidemiol. 2024 Nov 22;39(11):1267–76. doi: 10.1007/s10654-024-01171-z (PMC11646271; doi:10.1007/s10654-024-01171-z)
Supplement: Supplementary file 1 — Supplementary Material 1 [file 10654_2024_1171_MOESM1_ESM.docx]

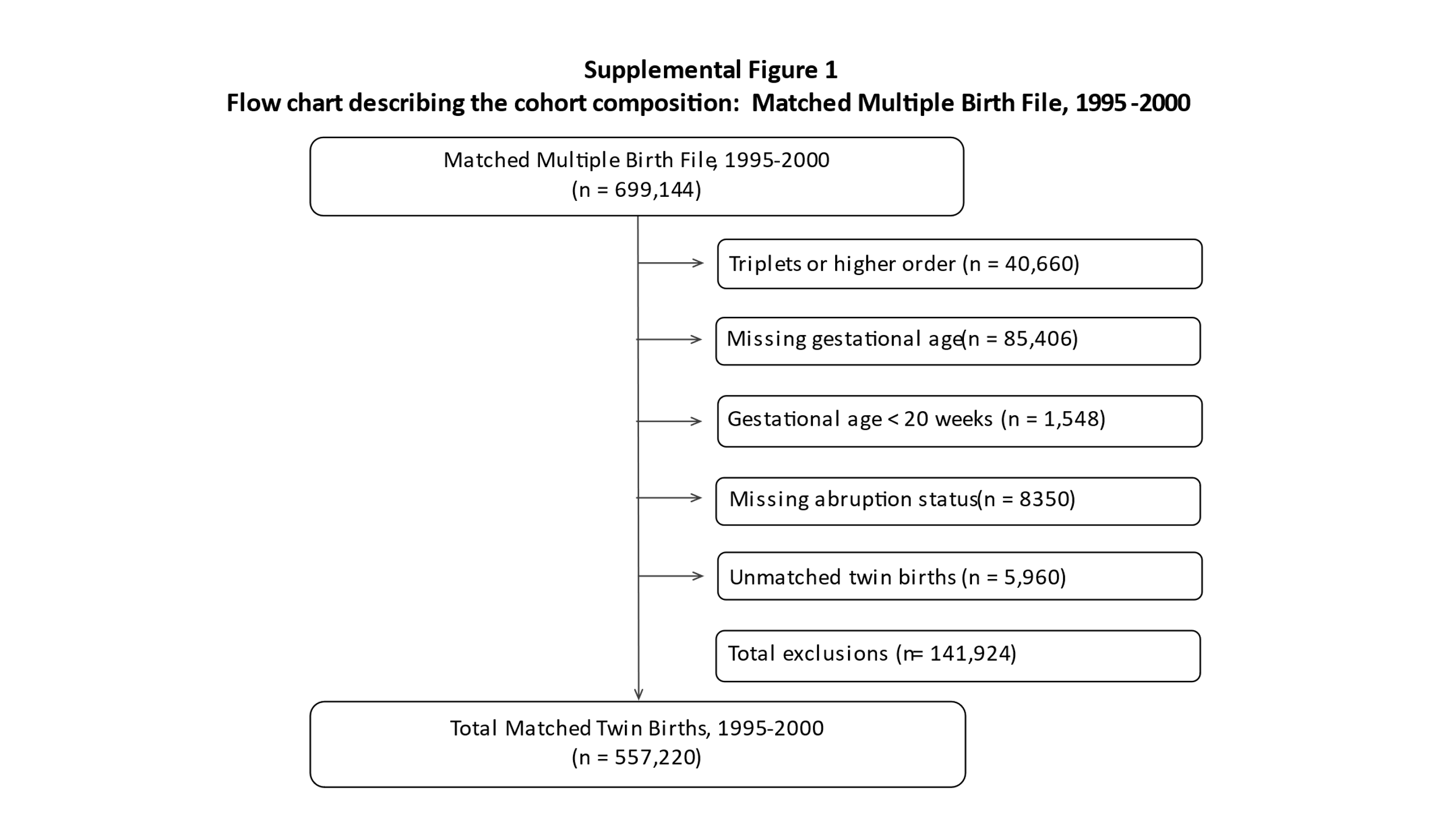


**Supplemental Figure 2**

**Cumulative percentage of deliveries among abruption and non-abruption births by gestational age, US Matched Multiple Birth File, 1995-2000**

**Supplemental Table 1**

**Distribution of maternal characteristics and risk factors for placental abruption among twin births:**

**US Matched Multiple Birth File, 1995-2000**

|  | **Total cohort** | | | **Placental abruption** | | |
| --- | --- | --- | --- | --- | --- | --- |
|  | **Number** | **col_%_** | **Number** | | **col_%_** | **row_%_** |
|  |  |  |  | |  |  |
| All births | 557 220 | 100.0 | 7032 | | 100.0 | 1.3 |
|  |  |  |  | |  |  |
| Maternal age, years |  |  |  | |  |  |
| <20 | 39 019 | 7.0 | 476 | | 6.8 | 1.2 |
| 20-24 | 107 310 | 19.3 | 1303 | | 18.5 | 1.2 |
| 25-29 | 150 854 | 27.1 | 1801 | | 25.6 | 1.2 |
| 30-34 | 158 270 | 28.4 | 2034 | | 28.9 | 1.3 |
| 35-39 | 84 734 | 15.2 | 1165 | | 16.6 | 1.4 |
| ≥40 | 17 033 | 3.1 | 253 | | 3.6 | 1.5 |
|  |  |  |  | |  |  |
| Parity |  |  |  | |  |  |
| 1 | 92 755 | 16.8 | 1058 | | 15.2 | 1.1 |
| 2 | 165 883 | 30.0 | 1883 | | 27.1 | 1.1 |
| ≥3 | 294 788 | 53.3 | 4007 | | 57.7 | 1.4 |
|  |  |  |  | |  |  |
| Maternal race/ethnicity |  |  |  | |  |  |
| White | 437 116 | 78.5 | 5537 | | 78.7 | 1.3 |
| Black | 102 613 | 18.4 | 1287 | | 18.3 | 1.3 |
| Other | 17 491 | 3.1 | 208 | | 3.0 | 1.2 |
|  |  |  |  | |  |  |
| Marital status |  |  |  | |  |  |
| Single | 153 585 | 27.7 | 2065 | | 29.8 | 1.3 |
| Married | 401 789 | 72.4 | 4871 | | 70.2 | 1.2 |
|  |  |  |  | |  |  |
| Mother’s education, years |  |  |  | |  |  |
| <9 | 17 352 | 3.2 | 188 | | 2.7 | 1.1 |
| 9-12 | 235 976 | 43.0 | 3155 | | 45.8 | 1.3 |
| 13-15 | 231 359 | 42.2 | 2795 | | 40.6 | 1.2 |
| ≥16 | 64 016 | 11.7 | 752 | | 10.9 | 1.2 |
|  |  |  |  | |  |  |
| Maternal smoking |  |  |  | |  |  |
| Smoker | 54 762 | 10.5 | 980 | | 15.3 | 1.8 |
| Non-smoker | 467 326 | 89.5 | 5435 | | 84.7 | 1.2 |
|  |  |  |  | |  |  |
| Chronic hypertension |  |  |  | |  |  |
| Present | 6031 | 1.1 | 111 | | 1.6 | 1.8 |
| Absent | 547 257 | 98.9 | 6854 | | 98.4 | 1.3 |
|  |  |  |  | |  |  |
| Preeclampsia/eclampsia |  |  |  | |  |  |
| Present | 49 208 | 8.9 | 527 | | 7.6 | 1.1 |
| Absent | 504 080 | 91.1 | 6438 | | 92.4 | 1.3 |
|  |  |  |  | |  |  |
| Diabetes |  |  |  | |  |  |
| Present | 19 800 | 3.6 | 251 | | 3.6 | 1.3 |
| Absent | 533 488 | 96.4 | 6714 | | 96.4 | 1.3 |
|  |  |  |  | |  |  |
| Child Sex |  |  |  | |  |  |
| Male | 280 589 | 50.4 | 3589 | | 51.1 | 1.3 |
| Female | 276 629 | 49.6 | 3442 | | 49.0 | 1.2 |
|  |  |  |  | |  |  |
| Matched sex |  |  |  | |  |  |
| Mixed sex | 185 811 | 33.4 | 2514 | | 35.8 | 1.4 |
| Both males | 187 685 | 33.7 | 2337 | | 33.2 | 1.3 |
| Both females | 183 724 | 33.0 | 2181 | | 31.0 | 1.2 |
|  |  |  |  | |  |  |
| Gestational age, weeks |  |  |  | |  |  |
| ≥37 | 241 759 | 43.4 | 1116 | | 15.9 | 0.5 |
| <37 | 315 461 | 56.6 | 5916 | | 84.1 | 1.9 |
| 34-36 | 200 252 | 35.9 | 1962 | | 27.9 | 1.0 |
| 32-33 | 48 505 | 8.7 | 1058 | | 15.1 | 2.2 |
| 28-31 | 36 932 | 6.6 | 1392 | | 19.8 | 3.8 |
| <28 | 29 772 | 5.3 | 1504 | | 21.4 | 5.1 |
|  |  |  |  | |  |  |

**Supplemental Table 2**

**Unadjusted hazard ratios and 95% CI of the association between placental abruption and mortality risk, with and without twin cluster sets, US Matched Multiple Birth File, 1995-2000**

|  | **Without Clustering** | **With Clustering** |
| --- | --- | --- |
|  | **Unadjusted HR (95% CI)** | **Unadjusted HR (95% CI)** |
|  |  |  |
| ***Preterm delivery <37 weeks*** | | |
| Stillbirth | 14.5 (10.0-21.0) | 14.5 (9.6-21.9) |
| Neonatal mortality | 2.9 (1.2-7.1) | 2.9 (1.2-7.1) |
| Perinatal mortality | 9.2 (6.6-12.9) | 9.2 (6.3-13.5) |
|  |  |  |
|  |  |  |
| ***Preterm delivery <34 weeks*** | | |
| Stillbirth | 9.8 (7.7-12.5) | 9.8 (7.5-12.9) |
| Neonatal mortality | 4.4 (2.9-6.6) | 4.4 (2.9-6.7) |
| Perinatal mortality | 7.4 (6.0-9.1) | 7.4 (5.9-9.3) |
|  |  |  |
|  |  |  |
| ***Preterm delivery <32 weeks*** | | |
| Stillbirth | 9.6 (7.9-11.6) | 9.6 (7.7-11.9) |
| Neonatal mortality | 4.0 (2.8-5.6) | 4.0 (2.8-5.7) |
| Perinatal mortality | 7.1 (6.0-8.4) | 7.1 (5.9-8.6) |
|  |  |  |
